# Anti-Human T-Cell Leukemia Virus Type 1 (HTLV-1) Antibody Assays in Cerebrospinal Fluid for the Diagnosis of HTLV-1-Associated Myelopathy/Tropical Spastic Paraparesis

**DOI:** 10.1128/JCM.03230-20

**Published:** 2021-04-20

**Authors:** Daisuke Kodama, Masakazu Tanaka, Toshio Matsuzaki, Satoshi Nozuma, Eiji Matsuura, Hiroshi Takashima, Shuji Izumo, Ryuji Kubota

**Affiliations:** a Division of Neuroimmunology, Joint Research Center for Human Retrovirus Infection, Kagoshima University, Kagoshima City, Japan; b Medical Corporation Sanshukai, Ohkatsu Hospital, Kagoshima City, Japan; c Department of Neurology and Geriatrics, Kagoshima University Graduate School of Medical and Dental Sciences, Kagoshima City, Japan; Rhode Island Hospital

**Keywords:** HTLV-1, antibody, HAM/TSP, CSF, PA, CLIA, CLEIA

## Abstract

The anti-human T-cell leukemia virus type 1 (HTLV-1) antibody assay in common use has changed from the particle agglutination (PA) method to chemiluminescent immunoassay (CLIA) and chemiluminescent enzyme immunoassay (CLEIA). These assays were validated in serum but not in cerebrospinal fluid (CSF). However, anti-HTLV-1 antibody positivity in CSF is a requisite for diagnosing HTLV-1-associated myelopathy/tropical spastic paraparesis (HAM/TSP). We qualitatively compared the assays in CSF from 47 HAM/TSP patients diagnosed using PA, 15 HTLV-1 carriers (HCs), and 18 negative controls. In determining the positivity or negativity of CSF anti-HTLV-1 antibodies, we used serum cutoff points for CLIA and CLEIA because CSF cutoff points had not been decided. Truth table analysis revealed that the performance of CLIA was closer to that of PA and that CLEIA had low sensitivity. CSF antibodies from HAM/TSP patients were all positive by PA and CLIA but 83.0% positive by CLEIA. CSF antibodies from HCs were positive in 73.3%, 80.0%, and 6.7% by PA, CLIA, and CLEIA, respectively. Receiver operator characteristic curve analysis for CSF revealed that with the default cutoff point used for serum, CLIA and PA had comparable performances and CLEIA was less sensitive. The best performances of CLIA and CLEIA with adjusted cutoff points were 94.8% sensitivity and 95.5% specificity and 89.7% sensitivity and 95.5% specificity, respectively. We conclude that low-sensitivity CLEIA can underdiagnose HAM/TSP and that CLIA is a better alternative to PA in anti-HTLV-1 antibody assay for CSF with the current cutoff points.

## Abstract (precis)

The anti-human T-cell leukemia virus type 1 (HTLV-1) antibody assay in common use has changed from the particle agglutination (PA) method to chemiluminescent immunoassay (CLIA) and chemiluminescent enzyme immunoassay (CLEIA). These assays were validated in serum but not in cerebrospinal fluid (CSF). However, anti-HTLV-1 antibody positivity in CSF is a requisite for diagnosing HTLV-1-associated myelopathy/tropical spastic paraparesis (HAM/TSP).

## INTRODUCTION

Human T-cell leukemia virus type 1 (HTLV-1) is a human retrovirus that causes adult T-cell leukemia (1–3), HTLV-1-associated myelopathy/tropical spastic paraparesis (HAM/TSP) (4–6), and other related diseases. HAM/TSP is a neurological disorder characterized by spastic paraparesis and urinary dysfunction. The WHO diagnostic criteria for HAM/TSP include the unique neurological symptoms, positive anti-HTLV-1 antibody (Ab) in both serum and cerebrospinal fluid (CSF), and exclusion of other diseases (7).

A screening assay for anti-HTLV-1 Ab was established in 1983 (8). The gelatin particle agglutination (PA) assay, which has high sensitivity and detects IgG and IgM to HTLV-1, became commercially available in 1986 (Serodia-HTLV-1; Fujirebio Inc., Tokyo, Japan) (9). The sensitivity and specificity of PA were 100% in 200 samples verified by an HTLV-1 serum panel (10). Therefore, PA has primarily been used as a reliable screening test for HTLV-1 (11). PA does not distinguish between HTLV-1 and HTLV-2 (12). The latter is suspected to cause hairy cell leukemia (13), and their relation to neurological disease is controversial (14). In Japan, HTLV-2 is not a health problem because HTLV-2 infection is extremely rare (15) and was not detected in HAM/TSP patients (16, 17). However, it is necessary to discriminate between the two types in some areas of the world where HTLV-2 infection rates are relatively high (18, 19). As a confirmatory test for anti-HTLV-1/HTLV-2 Ab, Western blotting often shows the intermediate state by the WHO criteria (20–22). Therefore, efforts have been made to create a better system for diagnosing HTLV-1 infection (23, 24). As an alternative confirmatory test, the line immunoassay (LIA) was developed to improve discrimination between HTLV-1 and HTLV-2 in serum (12, 25, 26).

Ab titer by PA is determined by macroscopic findings in serial dilutions comparing sensitized and unsensitized reactions. Recent automated Ab assays used in diagnosing HTLV-1 infection include chemiluminescent immunoassay (CLIA) and chemiluminescent enzyme immunoassay (CLEIA). The former utilizes acridinium ester-labeled mouse antibody to human IgG, and the latter uses alkaline phosphatase-labeled mouse antibody to human IgG. These two assays detect antibodies to p19, p21, and p24 of HTLV-1 (27–29). These automated assays are increasingly replacing PA. Anti-HTLV-1 Ab assays in serum are enough for the diagnosis of HTLV-1 infection and did not need to detect Ab in CSF. After discovery of HAM/TSP, positivity of CSF anti-HTLV-1 Ab became needed for diagnosis. However, there are several problems with CLIA and CLEIA in the diagnosis of HAM/TSP. First, anti-HTLV-1 Ab assays are needed for validation in CSF. For diagnosis of HAM/TSP, the serum examination protocol, including cutoff points for CLIA and CLEIA, was adopted for CSF examination and routinely accepted without validation. Therefore, the cutoff value or index settings for CSF Ab assays need to be considered. Second, supportive PCR to confirm CSF Ab assay results is not easy to perform. It requires more than 10 to 15 ml of CSF by lumbar puncture in addition to the volume for routine laboratory tests. Third, CSF Ab assays cannot distinguish dichotomously between HAM/TSP patients and HTLV-1 carriers (HCs). As CSF samples from one-fifth of HCs were previously reported positive, CSF anti-HTLV-1 Ab in HCs is not necessarily negative (30).

Therefore, we compared anti-HTLV-1 Ab assays in CSF to validate PA, CLIA, and CLEIA anti-HTLV-1 Ab assays for accurate HAM/TSP diagnosis.

## MATERIALS AND METHODS

### Subjects.

We used sets of serum, CSF, and peripheral blood mononuclear cell (PBMC) samples from 47 subjects with HAM/TSP, 15 HCs, and 18 negative controls (NCs), from whom written informed consent was obtained. The sample sets from a subject were obtained within a week, and the patients were not prescribed any anti-inflammatory drugs. Subjects suspected of adult T-cell leukemia or other HTLV-1-related diseases were excluded. HAM/TSP diagnosis was made when seropositive subjects fulfilled the WHO diagnostic criteria (7), including neurological symptoms, positivity for anti-HTLV-1 Ab in CSF by PA, and exclusion of other neurological disorders. HCs included 15 HTLV-1 carriers without inflammatory neurological diseases. HCs underwent lumbar puncture, and HAM/TSP was ruled out. NCs were the subjects from whom CSF were collected because they were suspected of noninflammatory neurological diseases but were later revealed to be HTLV-1 seronegative by PA. Serum and CSF samples were stocked at –20°C, and PBMCs were cryopreserved in liquid nitrogen until use. This study was conducted with the permission of the Ethics Committee of the Kagoshima University Hospital.

### Quantitative PCR assay for HTLV-1 provirus.

To confirm HTLV-1 infection, we extracted DNA from cryopreserved PBMCs and performed a quantitative PCR assay for HTLV-1 proviral load (HTLV-1 PVL) as previously described (31). The detection limit was 10 copies/10^4^ PBMCs, and subjects showing more than the limit were determined to be PCR positive and were diagnosed as infected with HTLV-1 (32). In this regard, the PCR positivity in PBMCs was qualitatively used as a confirmation of serum anti-HTLV-1 Ab positivity.

### Anti-HTLV-1 Ab assays and determination of anti-HTLV-1 Ab positivity in serum and CSF.

We measured anti-HTLV-1 Ab levels by PA, CLIA, and CLEIA. The cryopreserved serum or CSF in a tube was thawed, and the three assays were performed simultaneously. Serum anti-HTLV-1 Ab titer by PA (Serodia-HTLV-1; Fujirebio Inc., Tokyo, Japan) was measured according to the manufacturer’s protocol and interpretation criteria. Seropositivity (PA positivity in serum) was defined as a titer greater than 16× by PA (33), while anti-HTLV-1 Ab positivity in CSF was defined as a titer greater than 4×. This CSF criterion is due to a procedural limit of PA and was adopted to not exclude any HAM/TSP cases (34). Serum and CSF samples were assayed by CLIA (Architect-rHTLV-I/II; Abbott Laboratories, Chicago, IL) and CLEIA (Lumipulse-HTLV-I forte; Fujirebio, Tokyo, Japan) according to each kit’s protocol for serum because of the lack of CSF protocols. A serum or CSF sample was judged positive when the signal-to-cutoff ratio (S/CO) of the sample was greater than 1.0 by CLIA or when the cutoff index (COI) of the sample was greater than 1.0 by CLEIA. These cutoff values in CSF were adopted from those in serum.

### Definition of true positive for anti-HTLV-1 Ab in serum and CSF.

A true-positive anti-HTLV-1 Ab in serum (i.e., HTLV-1 infection) was defined as when both anti-HTLV-1 Ab in serum by PA and real-time PCR for HTLV-1 PVL were positive. On the other hand, PA positivity in CSF was expediently regarded as true when PA showed a titer of 4× or more without PCR because of the difficulty to collect enough DNA from a CSF sample.

### Truth table analysis of anti-HTLV-1 Ab assays.

Truth table analysis was performed to evaluate the similarity of performance (sensitivity and specificity) of Ab assays (CLIA or CLEIA) to that of PA when PA results were regarded as true. The performance of anti-HTLV-1 Ab assays in serum and CSF was calculated by 2 × 2 truth tables. For serum, PA-positive or PA-negative samples included those from HAM/TSP patients and HCs or those from NCs, respectively. For CSF, PA-positive samples included those from HAM/TSP patients and HCs with PA-positive CSF (HC-CSF^+^). PA-negative samples were those from HCs with PA-negative CSF (HC-CSF^−^) and NCs. Additionally, we investigated CSF samples for the consistency of the Ab results by the three assays.

### ROC curve analysis.

Receiver operating characteristic (ROC) curve analysis was performed to predict the best potential performance of CLIA or CLEIA for serum and CSF from HAM/TSP patients, HCs, and NCs under the situation in which the cutoff points could be modified. We calculated the maximum Youden index (sensitivity + specificity − 1) for each ROC curve to determine the best cutoff points.

### Logarithmic transformation of anti-HTLV-1 Ab data in serum and CSF.

The anti-HTLV-1 Ab levels were logarithmically transformed to bring their distribution closer to a normal distribution. Raw data for PA titer were transformed into a 2-based logarithm, and those data by CLIA (S/CO) or CLEIA (COI) were transformed into a 10-based logarithm. The base 2 of PA corresponds to the serial 2-fold dilution protocol. PA Ab titers below 16× in serum and those below 4× in CSF could not be measured because of the actual procedure, and those logarithmically transformed values were assigned as 0. Similarly, since the detection limits of CLIA and CLEIA were 0.1, the transformed values of Ab data below 0.1 from these assays were assigned as −1.

### Comparison and correlation analysis of anti-HTLV-1 Ab levels in serum and CSF.

Anti-HTLV-1 Ab levels were compared between serum and CSF and between HCs and HAM/TSP patients. A correlation analysis was performed to assess the consistency of anti-HTLV-1 Ab levels among assays.

### Correlation analysis between anti-HTLV-1 Ab levels and HTLV-1 PVL.

A correlation analysis was conducted between either serum or CSF anti-HTLV-1 Ab levels measured by the three assays and HTLV-1 PVL in PBMCs.

### Statistics.

We compared the ages of sample groups by one-way analysis of variance (ANOVA). We performed a two-tailed Mann-Whitney U test with Bonferroni correction to compare Ab levels, Spearman correlation analysis, and simple linear regression analysis. A *P* value of less than 0.05 was considered statistically significant. ROC curve analysis was performed using GraphPad Prism 6.0 (GraphPad Software, Inc., La Jolla, CA). Statistics were performed using Statcel version 3.0 (OMS Publishing, Inc., Tokorozawa, Saitama, Japan) or GraphPad Prism 6.0.

## RESULTS

### Serum anti-HTLV-1 Ab positivity was matched by all assays.

The characteristics of subjects for each group, including diagnosis, sex, age, and PVL, are summarized in Table 1. Sixty-two serum samples from HAM/TSP patients and HCs were positive and 18 samples from NCs were negative by all assays. All seropositive subjects showed PVLs of more than 10 copies/10^4^ PBMCs, and all seronegative subjects had negative PCR for HTLV-1 provirus. All assays matched the Ab results (positive or negative) in serum.

**TABLE 1:** Summary of subjects^*a*^

Parameter	Value for subjects with indicated diagnosis		
	HAM/TSP	HC	NC
No. of subjects	47	15	18
Sex (male/female)	12/35	6/9	10/8
Age (yrs)^*b*^	62.8 ± 11.7^*c*^	69.9 ± 11.4	49.8 ± 19.5
Anti-HTLV-1 antibody in serum^*d*^
Log_2_ titer by PA	14.0 ± 2.0	10.2 ± 2.5	ND
Log_10_ S/CO by CLIA	2.07 ± 0.12	1.97 ± 0.16	ND
Log_10_ COI by CLEIA	2.1 ± 0.3	1.4 ± 0.1	ND
Anti-HTLV-1 antibody in CSF^*d*^
Log_2_ titer by PA	6.7 ± 2.5	2.4 ± 0.5	ND
Log_10_ S/CO by CLIA	1.36 ± 0.41	0.43 ± 0.18	ND
Log_10_ COI by CLEIA	0.4 ± 0.6	−0.7 ± 0.1	ND
HTLV-1 PVL (copies/10^4^ PBMCs)^*e*^	1,138.5 ± 972.9	162.9 ± 129.4	ND

a The cutoff points for serum were a titer of 16×, S/CO of 1.0, and COI of 1.0 for PA, CLIA, and CLEIA, respectively. Those for CSF were a titer of 4×, S/CO of 1.0, and COI of 1.0 for PA, CLIA, and CLEIA, respectively. The cutoff points in CLIA and CLEIA for CSF were adopted from those in serum and were commonly used because cutoff points for CSF had not been determined. Diagnosis of HTLV-1-associated myelopathy/tropical spastic paraparesis (HAM/TSP) was made according to WHO diagnostic criteria. Seropositive subjects who did not satisfy the diagnostic criteria were diagnosed as HTLV-1 carriers (HCs). Seronegative subjects were diagnosed as negative controls (NCs). ND, not detected.

b There was a significant difference in age among the three groups by one-way ANOVA (*P *< 0.01).

c The values are expressed as means ± standard deviations.

d Anti-HTLV-1 antibodies were logarithmically transformed from raw data by each assay.

e There was a significant difference in PVL between HAM/TSP patients and HCs by the Mann-Whitney U test (*P *= 0.02).

### The truth table for CSF Ab positivity showed that CLIA was close to PA.

Using the qualitative data regarding positive or negative anti-HTLV-1 Ab, we conducted truth table analysis to investigate whether CLIA or CLEIA performed more closely to PA. In serum, sensitivity and specificity were 100% for CLIA and CLEIA because the positive or negative results were completely the same among the three assays (data not shown). Under the assumption that CSF PA results were true, there were 58 true-positive CSF samples (47 HAM/TSP and 11 HC-CSF^+^) and 22 true-negative CSF samples (4 HC-CSF^−^ and 18 NCs) (Table 2). CLIA showed 96.6% sensitivity in discriminating PA-positive from PA-negative samples, while CLEIA had 69.0% sensitivity. CLEIA, however, showed better specificity than CLIA, at 100.0% versus 86.4%, respectively.

**TABLE 2:** Performance of anti-HTLV-1 Ab assays in CSF by CLIA and CLEIA compared with PA

Assay	Result	Anti-HTLV-1 Ab in CSF^*a*^		Total^*c*^
		PA positive (HAM/TSP and HC-CSF^+^)	PA negative (HC-CSF^−^ and NC^*b*^)	
CLIA	Positive	56	3	59
	Negative	2	19	21
	Total^*d*^	58 (sensitivity, 96.6% [56/58])	22 (specificity, 86.4% [19/22])	80
CLEIA	Positive	40	0	40
	Negative	18	22	40
	Total^*d*^	58 (sensitivity, 69.0% [40/58])	22 (specificity, 100.0% [22/22])	80

a In anti-HTLV-1 antibody (Ab) assay in CSF, PA-positive or PA-negative results were regarded as true-positive or true-negative results, respectively. HC-CSF^+^, HC with PA-positive CSF; HC-CSF^−^, HC with PA-negative CSF.

b All CSF samples from NCs (*n* = 18) were negative in all assays.

c The total number represents the sum of the numbers of PA-positive and PA-negative samples in the same row.

d The total number represents the sum of the numbers of PA-positive and PA-negative samples in the same column.

### Inconsistency in CSF anti-HTLV-1 Ab positivity among the assays.

CSF samples from all HAM/TSP patients showed anti-HTLV-1 Ab positivity at 100.0% (47/47) by PA and CLIA but 83.0% (39/47) by CLEIA. Those from all HCs were positive at 73.3% (11/15) by PA, 80.0% (12/15) by CLIA, and 6.7% (1/15) by CLEIA. CSF samples from NCs were negative by all assays. Next, we focused on the inconsistent results among the three assays as shown in Table 3. We excluded 2 of 15 HCs and 39 of 47 HAM/TSP patients from Table 3 because they showed consistent positive or negative results. One of the excluded HCs showed all positive results (titer of 64×, S/CO of 52.76, and COI of 1.4), and the other showed all negative (titer of <4×, S/CO of 0.38, and COI of <0.1). All of the excluded 39 HAM/TSP patients were positive in the three assays.

**TABLE 3:** Inconsistency of CSF anti-HTLV-1 Ab results among assays

Diagnosis	Sample no.	PA		CLIA		CLEIA	
		Titer	Result^*a*^	S/CO	Result	COI	Result
HAM/TSP	1	32×	+	8.25	+	0.3	–
2	32×	+	6.93	+	0.2	–
3	32×	+	2.00	+	0.2	–
4	16×	+	7.09	+	0.3	–
5	8×	+	9.02	+	0.4	–
6	8×	+	5.57	+	0.2	–
7	8×	+	4.18	+	0.8	–
8	8×	+	2.49	+	0.1	–
HC	1	64×	+	33.07	+	0.7	–
2	8×	+	10.44	+	0.2	–
3	8×	+	4.97	+	0.1	–
4	8×	+	4.73	+	0.4	–
5	8×	+	4.67	+	0.7	–
6	8×	+	1.52	+	0.1	–
7	8×	+	0.77	–	0.1	–
8	4×	+	2.03	+	0.2	–
9	4×	+	1.16	+	0.1	–
10	4×	+	0.13	–	0.1	–
11	<4×	–	5.33	+	0.2	–
12	<4×	–	1.49	+	0.1	–
13	<4×	–	1.24	+	0.1	–

a Shown are CSF assay results inconsistent among assays. A plus or minus represents a positive or negative result in each assay. The results of CSF samples were inconsistent in 8 of 47 samples from HAM/TSP patients and 13 of 15 samples from HCs. The remaining 39 HAM/TSP patients with consistent results showed all positive. In the remaining 2 HCs with consistent results, one showed all positive (titer of 64×, S/CO of 52.76, and COI of 1.4), and the other showed all negative (titer of <4×, S/CO of 0.38, and COI of <0.1).

In the table of inconsistency, CSF samples from 17.0% (8/47) of HAM/TSP patients and 86.7% (13/15) of HCs showed inconsistent Ab results among the three assays (Table 3). The 13 inconsistent samples of HCs were negative by CLEIA, with a COI of 0.8 or less. All inconsistent CSF samples from HAM/TSP patients were positive by PA and CLIA but negative by CLEIA. In inconsistent CSF samples from HCs, two samples (HC 7 and 10) were positive by PA but negative by CLIA, and three samples (HC 11 to 13) were negative by PA but positive by CLIA. In some individuals with PA titers less than 32×, the Ab results were inconsistent with those by CLIA or CLEIA.

### ROC curve analysis revealed a closer performance of CLIA to that of PA in CSF.

**FIG 1:**
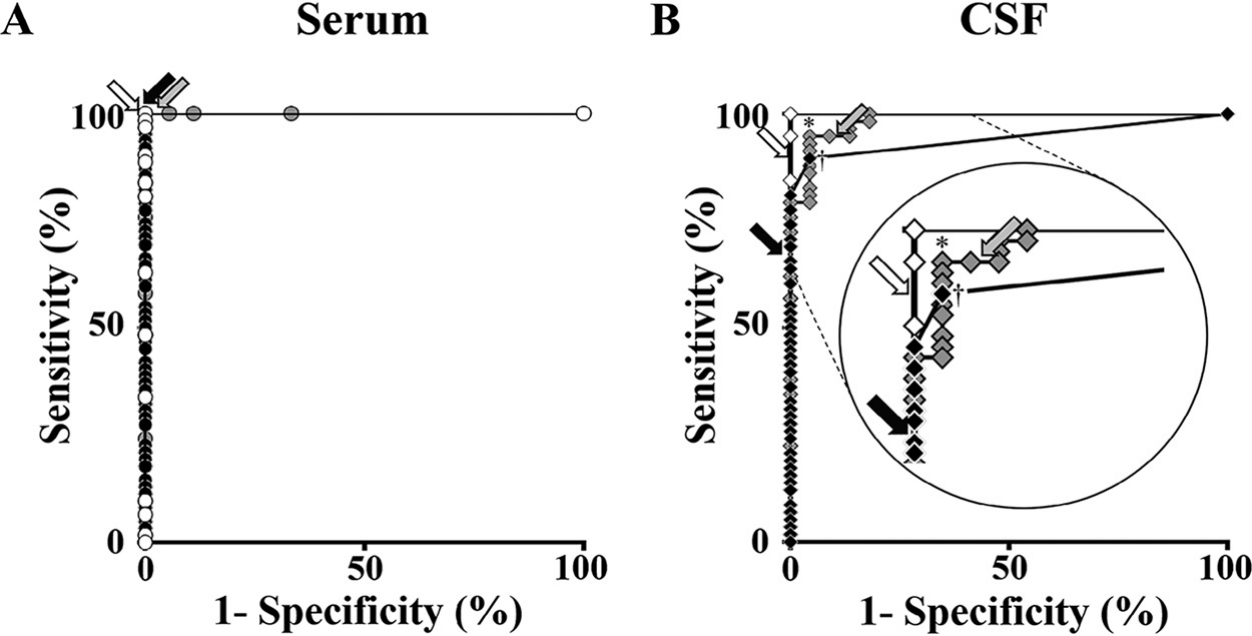
Receiver operator characteristic (ROC) curve analyses of anti-HTLV-1 Ab data in serum (A) and CSF (B) to determine PA-positive and PA-negative samples. All samples from HAM/TSP patients, HCs, and NCs were categorized into PA positive and PA negative. In panel B, the upper left corner of the plot is displayed with enlargement. White, gray, and black circles in panel A represent performances of PA, CLIA, and CLEIA in serum, respectively. White, gray, and black diamonds in panel B represent those of PA, CLIA, and CLEIA in CSF, respectively. The asterisk and dagger in panel B represent the cutoff point where the Youden index (sensitivity + specificity – 1) is maximal in CLIA and CLEIA, respectively. White, gray, and black arrows represent the default cutoff points of PA, CLIA, and CLEIA, respectively.

To determine which of CLIA and CLEIA performed more closely to PA, we conducted ROC curve analyses of anti-HTLV-1 Ab to establish PA-positive (true positive) and PA-negative (true negative) categories for serum and CSF. For serum, PA-positive and PA-negative categories included assay results from HAM/TSP patients and HCs and those from NCs, respectively. For CSF, PA-positive and PA-negative categories included assay results from HAM/TSP patients and HC-CSF^+^ and those from HC-CSF^−^ and NCs, respectively. The areas under the curves (AUCs) in serum by all assays were 1.0 (Fig. 1A). Meanwhile, in CSF, AUC by CLIA (0.985) was greater than that by CLEIA (0.944) (*P < *0.05), indicating that CLIA performed more closely to PA (Fig. 1B). These data supported the results of the truth table analysis (Table 2).

### The best cutoff points improved the assay performances in CSF.

Next, we determined the best cutoff points by maximum Youden index. CLIA showed the best potential performance in CSF, with 94.8% sensitivity and 95.5% specificity by the cutoff S/CO of 1.50 (asterisk in Fig. 1B). Meanwhile, CLEIA showed 89.7% sensitivity and 95.5% specificity by the cutoff COI of 0.14 (dagger in Fig. 1B). The likelihood ratios (sensitivity/1 − specificity) of these best cutoff points were similar (CLIA, 21.1; CLEIA, 19.9). In determining anti-HTLV-1 Ab positivity or negativity in CSF, the best cutoff points improved assay performances of CLIA and CLEIA over the default cutoff points (Table 4). For CLIA, the sensitivity was almost the same, but the specificity and accuracy were slightly increased. For CLEIA, although the specificity was decreased, the sensitivity and accuracy were considerably increased.

**TABLE 4:** Performance improvement of anti-HTLV-1 Ab assays for CSF by the best cutoff point

Performance characteristic	CLIA performance at indicated cutoff point		CLEIA performance at indicated cutoff point	
	S/CO of 1.00 (default, adopted from serum)	S/CO of 1.50 (best^*a*^)	COI of 1.00 (default, adopted from serum)	COI of 0.14 (best^*a*^)
Sensitivity (%)	96.6	94.8	69.0	89.7
Specificity (%)	86.4	95.5	100.0	95.5
Accuracy (%)^*b*^	93.8	95.0	77.5	91.3
Likelihood ratio^*c*^	7.1	21.1	NA^*d*^	19.9

a The best cutoff points for anti-HTLV-1 Ab assay in CSF were determined by ROC curve analysis (Fig. 3B).

b Accuracy was calculated as (true positive + true negative)/(total number).

c Likelihood ratio was defined as sensitivity/(1 − specificity).

d NA, not applicable because the specificity of CLEIA at the default cutoff point was 100%.

### Anti-HTLV-1 Ab levels were lower in CSF than in serum from HAM/TSP patients and HCs.

**FIG 2:**
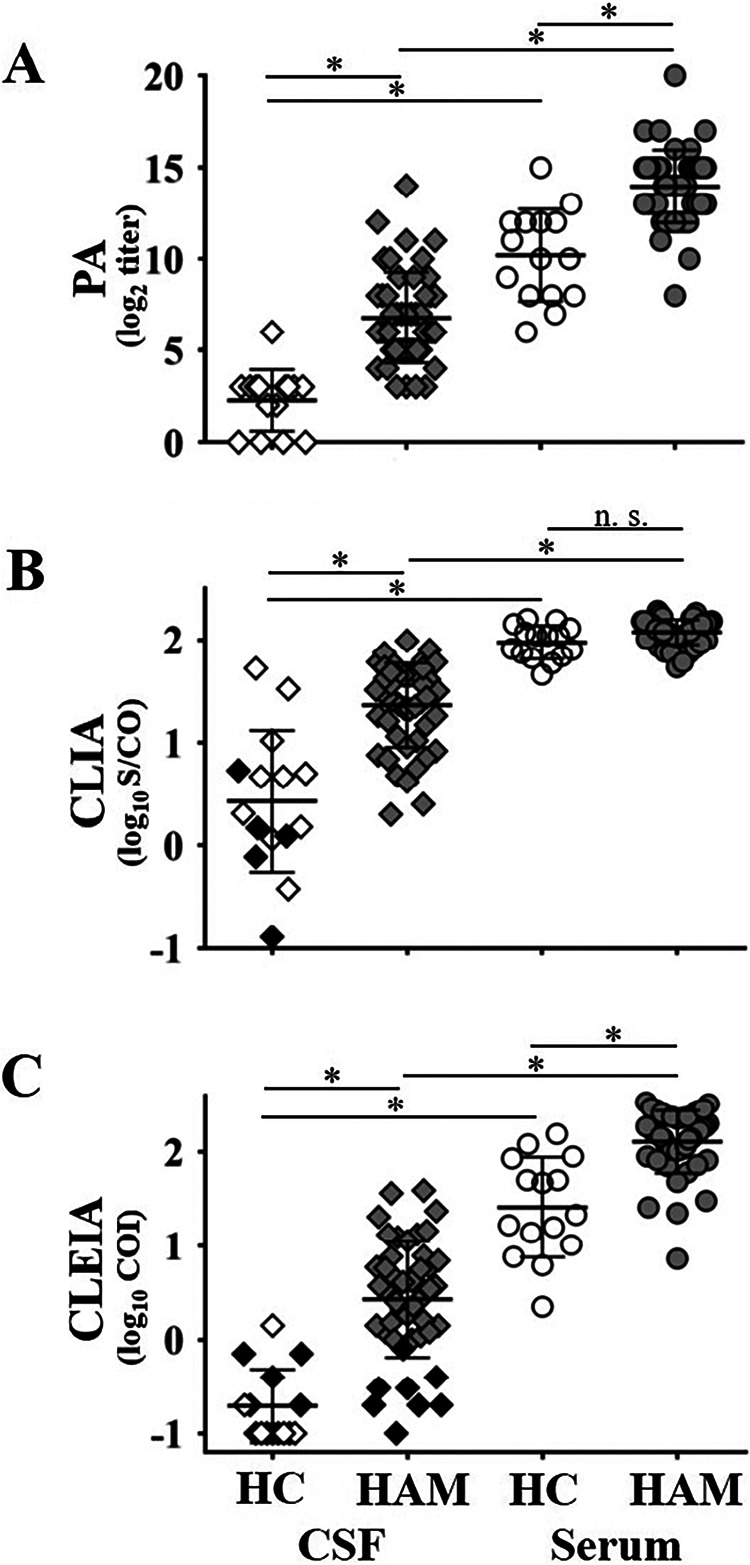
Anti-HTLV-1 Ab levels in serum and CSF from 47 HAM/TSP patients and 15 HCs by PA (A), CLIA (B), and CLEIA (C). Bars of each sample group represent means ± standard deviations. An asterisk over the bar indicates a *P *value of <0.01. Gray and white circles represent anti-HTLV-1 Ab levels in serum from HAM/TSP patients and HCs, respectively. Gray and white diamonds represent those in CSF from HAM/TSP patients and HCs, respectively. Black diamonds of CSF samples in CLIA or CLEIA represent the results (positive or negative) inconsistent with those of PA. n.s., not significant.

After evaluating Ab positivity or negativity as a qualitative analysis, we compared anti-HTLV-1 Ab levels as a quantitative analysis. Serum and CSF Ab levels were significantly higher in HAM/TSP patients than in HCs except for serum by CLIA (Fig. 2). Additionally, Ab levels were substantially lower in CSF than in serum in HAM/TSP patients and HCs (Fig. 2). In Fig. 2B and C, we colored the diamonds black when CSF samples in CLIA or CLEIA showed results (positive or negative) inconsistent with those in PA. The Ab levels of mismatched samples were distributed in a low range (black diamonds in Fig. 2B and C).

### Ab levels were strongly correlated among assays in CSF.

**FIG 3:**
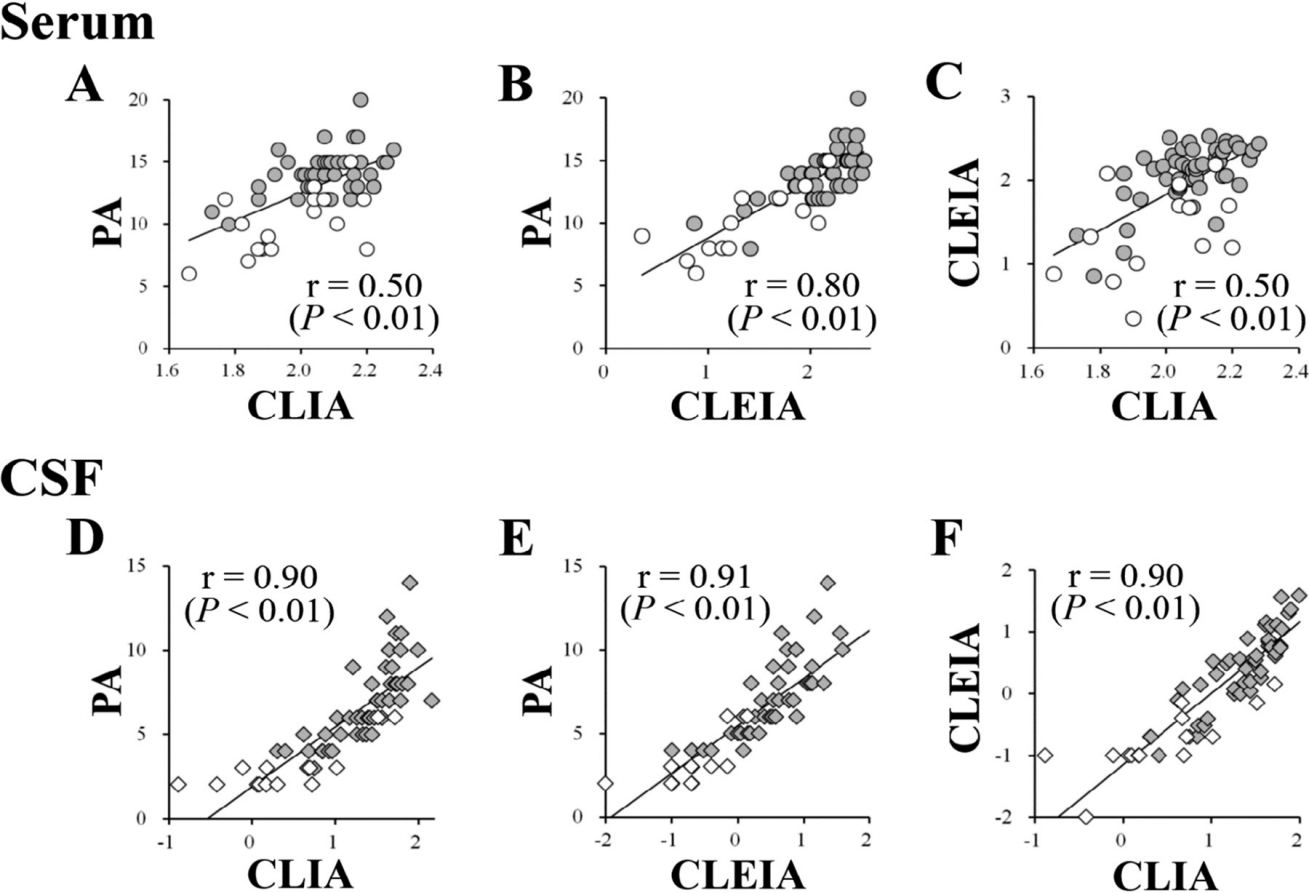
Correlation of anti-HTLV-1 Ab levels by PA, CLIA, and CLEIA in serum or CSF. Upper plots show the correlations of anti-HTLV-1 Ab levels in serum between PA and CLIA (A), PA and CLEIA (B), and CLIA and CLEIA (C). Lower plots show the correlations of anti-HTLV-1 Ab levels in CSF between PA and CLIA (D), PA and CLEIA (E), and CLIA and CLEIA (F). Gray and white circles represent anti-HTLV-1 Ab levels in serum from HAM/TSP patients and HCs, respectively. Gray and white diamonds represent those in CSF from HAM/TSP patients and HCs, respectively. The vertical and transverse axes represent logarithmically transformed anti-HTLV-1 Ab levels. The line represents a simple linear regression of anti-HTLV-1 Ab levels between assays. *R* and *P* values in each plot represent Spearman’s rank correlation coefficient and the significance level of Spearman correlation analysis, respectively.

At first, we tried logarithmically transformed anti-HTLV-1 Ab levels to standardize (35, 36). However, the data did not shape Gaussian distribution by histogram plots using either all samples, clinical categories (HAM/TSP or HCs), or sample source categories (serum or CSF) (data not shown). Therefore, we investigated the correlation among assays using the log-transformed raw data and nonparametric testing (Fig. 3). In serum Ab levels from HAM/TSP patients and HCs, PA and CLIA, PA and CLEIA, and CLEIA and CLIA showed relatively strong or strong correlations, with Spearman’s rank correlation coefficients of 0.50, 0.80, and 0.50, respectively (*P < *0.01 [Fig. 3A to C]). In CSF Ab data, the assays showed strong correlations, with correlation coefficients of 0.90, 0.91, and 0.90, respectively (*P < *0.01 [Fig. 3D to F]).

### Correlation between anti-HTLV-1 Ab levels and HTLV-1 PVL.

**FIG 4:**
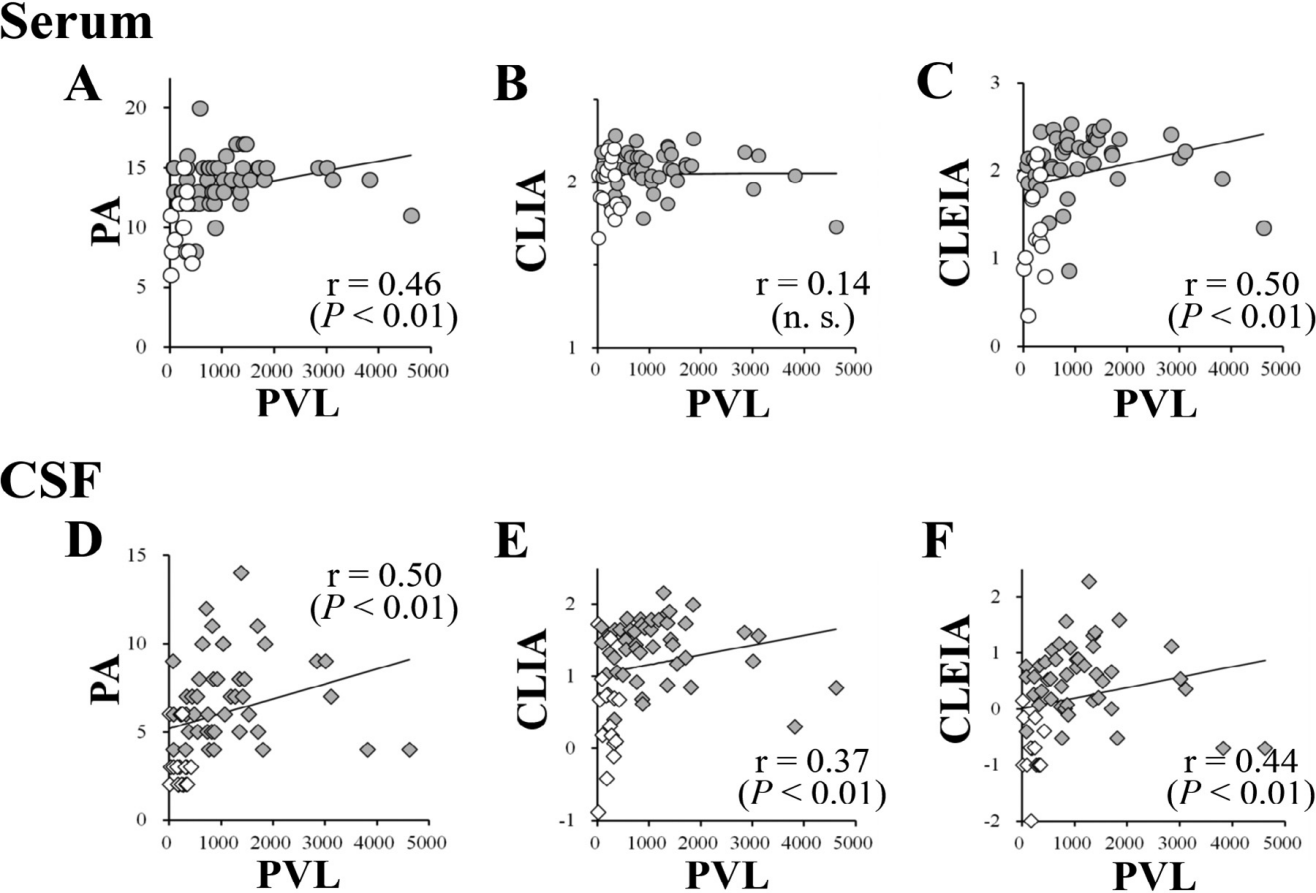
Correlation of HTLV-1 proviral load (PVL) and anti-HTLV-1 Ab levels by PA, CLIA, and CLEIA in serum or CSF. Upper plots show the correlations of anti-HTLV-1 Ab levels in serum by PA and PVL (A), CLIA and PVL (B), and CLEIA and PVL (C). Lower plots show the correlations of anti-HTLV-1 Ab levels in CSF by PA and PVL (D), CLIA and PVL (E), and CLEIA and PVL (F). Gray and white circles represent anti-HTLV-1 Ab levels in serum from HAM/TSP patients and HCs, respectively. Gray and white diamonds represent those in CSF from HAM/TSP patients and HCs, respectively. The vertical axis represents logarithmically transformed anti-HTLV-1 Ab levels in PA (log_2_ titer), CLIA (log_10_ S/CO), and CLEIA (log_10_ COI). The transverse axis represents HTLV-1 PVL in peripheral blood (copies/10^4^ PBMCs). The line represents a simple linear regression between anti-HTLV-1 Ab levels and HTLV-1 PVL. *R* and *P* values in each plot represent Spearman’s rank correlation coefficient and the significance level of Spearman correlation analysis, respectively.

PVL in HAM/TSP patients and HCs were 1,138.5 ± 972.9 and 162.9 ± 129.4 (mean ± standard deviation) copies/10^4^ PBMCs, respectively (Table 1). The PVL in HAM/TSP patients was 7-fold higher than that in HCs (*P* < 0.01). We investigated the relationship between serum or CSF anti-HTLV-1 Ab levels and HTLV-1 PVL in PBMCs (Fig. 4). The serum anti-HTLV-1 Ab levels by PA and CLEIA, but not CLIA, positively correlated with PVL. In CSF, the Ab levels measured by all assays showed positive correlations with PVL.

## DISCUSSION

Anti-HTLV-1 Ab assays have been validated in serum but not in CSF because it was enough to examine the Ab in serum to know whether subjects were infected with HTLV-1. However, after the discovery of HAM/TSP, it became necessary to determine whether CSF Ab is positive or negative for the diagnosis. We qualitatively validated anti-HTLV-1 Ab assays in CSF samples from HAM/TSP patients, HCs, and NCs. When determining anti-HTLV-1 Ab CSF positivity or negativity using serum cutoff points, the truth table analysis revealed that CLIA in CSF had a well-balanced performance. The assay showed 96.6% sensitivity and 86.4% specificity; however, CLEIA had 69.0% sensitivity and 100.0% specificity (Table 2). Therefore, CLIA is a more reliable and qualitative assay for detecting anti-HTLV-1 Ab in CSF. Thus, CLIA is a better alternative assay for PA to diagnose HAM/TSP.

The low sensitivity of CLEIA has a crucial impact on HAM/TSP diagnosis, given that anti-HTLV-1 Ab positivity in CSF is a requisite for diagnosis. In other words, assays with low sensitivity may exclude HAM/TSP cases with low Ab levels in CSF. The qualitative Ab assay results were all consistent in serum by all methods. However, there were inconsistent results in 21 CSF samples: all were negative by CLEIA (Table 3). A total of 18 of 62 PA-positive CSF samples were negative by CLEIA. Furthermore, 8 of 47 CSF samples from HAM/TSP patients were negative by CLEIA, indicating that 17.0% of HAM/TSP cases will be misdiagnosed as HCs. Thus, CLEIA for CSF using the default cutoff point is inappropriate for diagnosing HAM/TSP.

AUCs in the ROC curve analysis supported the truth table analysis results, indicating that CLIA for CSF is better than CLEIA to determine anti-HTLV-1Ab positivity at the default cutoff point (Fig. 1). On the other hand, the maximum Youden index in the ROC curve analysis indicated that the best potential performance of CLIA and CLEIA with the best cutoff points considerably resolves the inconsistency of anti-HTLV-1 Ab positivity in CSF among the assays. To correct inconsistent CSF results by CLIA and the low sensitivity of CLEIA, the cutoff points should be adjusted for the best performance. After determining the best cutoff points, we compared the performance regarding positive or negative CSF anti-HTLV-1 Ab (Table 4). The best cutoff points in CLIA and CLEIA improved the performance over the default cutoff points. Finally, they improved the sensitivity of CLEIA for HAM/TSP diagnosis from 69.0% to 89.7%, though the sensitivities of CLIA were almost the same, at 96.9% to 94.8% (Table 4). These results indicated that CLEIA with the best cutoff point was better suited for HAM/TSP diagnosis than that with the default cutoff point, though not as much as CLIA.

Although PA titer was measured macroscopically, the values in CLIA and CLEIA were calculated as ratios of measured values of specimens to those of the standards. Therefore, the difference in diluents and dilution ratios of specimens among assays probably did not affect the comparison between the assays. As a quantitative analysis, we compared Ab levels measured by the three assays. We found that HAM/TSP patients had higher anti-HTLV-1 Ab levels in serum and CSF than HCs except for serum by CLIA (Fig. 2). However, some of the Ab levels in CSF overlapped between HAM/TSP patients and HCs, which was also seen in serum (Fig. 2). These indicate that Ab levels in either serum or CSF cannot distinguish the two clinical statuses. CLIA shaped clusters in the serum Ab from HCs and HAM/TSP patients and did not show a significant difference between both (Fig. 2B). These suggest that the linearity of CLIA may be different from those of PA or CLEIA at high Ab levels, but the reason is unclear.

For HAM/TSP patients, anti-HTLV-1 Ab levels in CSF were lower than those in serum (Fig. 2). The finding is accepted by neurologists but has not been generally considered (37, 38). We confirmed it statistically in 47 HAM/TSP patients. The Ab in CSF consists of passive transfer and intrathecal synthesis. In previous studies, increased intrathecal IgG and anti-HTLV-1 Ab indices suggested that some anti-HTLV-1 Abs are produced in the central nervous system in HAM/TSP patients (37, 38). We did not aim to investigate the origin of anti-HTLV-1 Ab in CSF; therefore, we did not collect enough data, including albumin or IgG, for serum and CSF. These are needed to calculate the IgG index, which indicates newly synthesized Ab in the central nervous system. In addition to the intrathecal IgG synthesis, passive transfer of the Ab from serum to CSF may be another mechanism affecting Ab levels in CSF because Ab levels in serum were higher than in CSF.

Anti-HTLV-1 Ab was regarded as showing true positivity in this study when both serum anti-HTLV-1 Ab by PA and PCR for HTLV-1 were positive, indicating that the subject was infected with the virus. All seropositive subjects showed PVLs of more than 10 copies/10^4^ PBMCs, and all seronegative subjects showed no provirus. Diagnosis of HTLV-1 infection needs both a serum anti-HTLV-1 Ab screening test (PA, CLIA, or CLEIA) and a confirmatory test such as Western blotting or PCR. On the other hand, the diagnosis of HAM/TSP requires CSF anti-HTLV-1 Ab positivity in HTLV-1-infected individuals but not PCR positivity. Therefore, we detected CSF anti-HTLV-1 Ab PA titers over 4× but did not perform HTLV-1 PCR as a confirmatory test for anti-HTLV-1 Ab in CSF. Detection of HTLV-1 provirus in CSF may be difficult because HTLV-1 is usually detected in one of many cells, and PCR requires a large number of CSF cells. For example, even if we use a maximum of 10 ml of CSF for PCR, the total number of cells is limited to an order of magnitude of 10^4^. When the number of HTLV-1 provirus is fewer than 10 copies in the CSF sample, detection of HTLV-1 provirus can fail by the quantitative PCR with a detection limit of 10 copies/10^4^ cells. A study reported that PCR detected no PVL in CSF from subjects with serum Ab less than 64× (39). A method that is more sensitive and easier will be needed for confirmation of CSF anti-HTLV-1 Ab positivity.

We randomly recruited HAM/TSP patients regardless of the severity of the disease. Although serum anti-HTLV-1 Ab levels are not different between HAM/TSP patients with either rapid or slow progression, patients with rapid progression have higher Ab levels in CSF than those with slow progression (40). As a result of a random selection of the patients, our study did not include HAM/TSP patients with rapid progression due to the low frequency of this subtype. We collected blood and CSF samples once from subjects because ani-HTLV-1 Ab levels in serum remain stable during the course in HAM/TSP patients and HCs (41).

We recruited 47 HAM/TSP patients, 15 HCs, and 18 NCs for this study, and the sample size of each group was small. Also, our samples did not shape Gaussian distribution, probably due to the small size of the samples. We selected HAM/TSP patients who were not treated at the sampling; therefore, the number of HAM/TSP patients almost reached the maximum in our cohort. Another problem is that the numbers of HCs and NCs were small compared to that of the HAM/TSP group. However, we analyzed both serum and CSF from the subjects, and it was difficult to recruit more HCs and NCs because lumbar puncture to obtain CSF is somewhat harmful. We could not recruit any individuals without neurological symptoms because conducting the examination on healthy subjects is an ethical problem. To increase the subject numbers, joint research with other facilities would be needed.

We conclude that low-sensitivity CLEIA can underdiagnose HAM/TSP in some cases and that CLIA is a better anti-HTLV-1 Ab assay for CSF with the current cutoff point as a substitute for PA.
